# Supplementary material for: The Cell Surface Proteome of Malignant Peripheral Nerve Sheath Tumors Reveals Therapeutic Targets
Source: bioRxiv. 2026 Mar 14:2026.03.11.711103. Preprint. [Version 1] doi: 10.64898/2026.03.11.711103 (PMC13060849; doi:10.64898/2026.03.11.711103)

bioRxiv preprint doi: https://doi.org/10.64898/2026.03.11.711103; this version posted March 14, 2026. The copyright holder for this preprint (which was not certified by peer review) is the author/funder, who has granted bioRxiv a license to display the preprint in perpetuity. It is made available under aCC-BY 4.0 International license.

Supplementary Figure 1

A

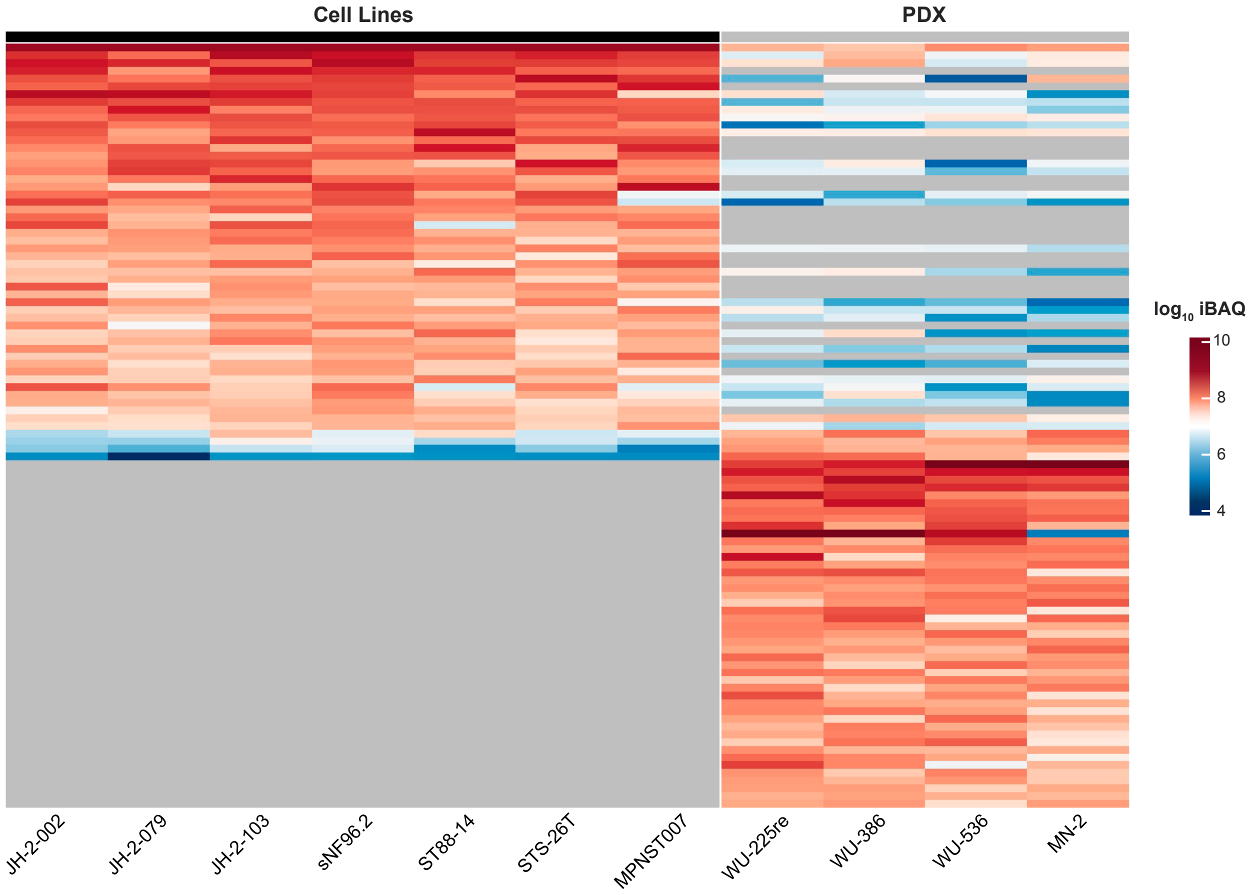

bioRxiv preprint doi: https://doi.org/10.64898/2026.03.11.711103; this version posted March 14, 2026. The copyright holder for this preprint (which was not certified by peer review) is the author/funder, who has granted bioRxiv a license to display the preprint in perpetuity. It is made available under aCC-BY 4.0 International license.

# Supplementary Figure 2

A

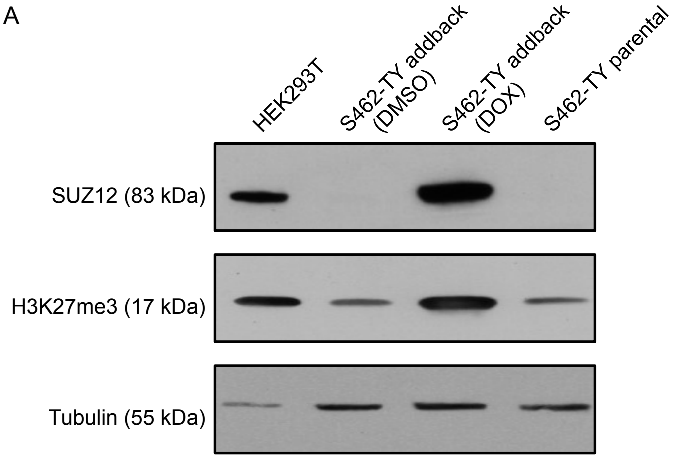

bioRxiv preprint doi: https://doi.org/10.64898/2026.03.11.711103; this version posted March 14, 2026. The copyright holder for this preprint (which was not certified by peer review) is the author/funder, who has granted bioRxiv a license to display the preprint in perpetuity. It is made available under aCC-BY 4.0 International license.

# Supplementary Figure 3

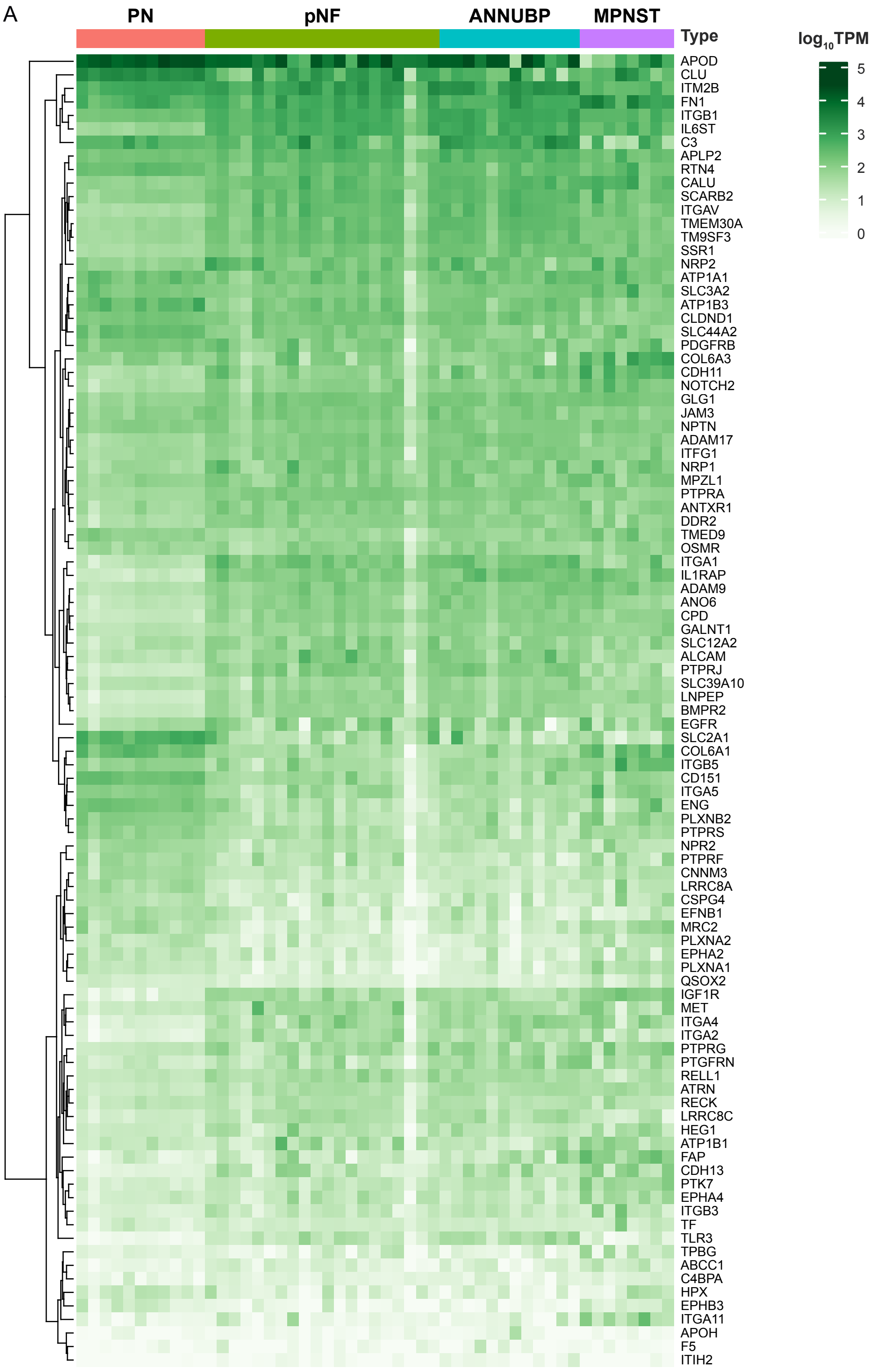

bioRxiv preprint doi: https://doi.org/10.64898/2026.03.11.711103; this version posted March 14, 2026. The copyright holder for this preprint (which was not certified by peer review) is the author/funder, who has granted bioRxiv a license to display the preprint in perpetuity. It is made available under aCC-BY 4.0 International license.

Supplementary Figure 4

A

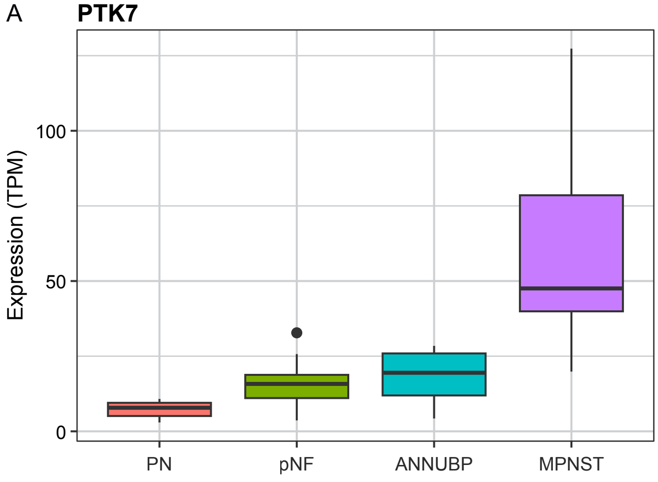

bioRxiv preprint doi: https://doi.org/10.64898/2026.03.11.711103; this version posted March 14, 2026. The copyright holder for this preprint (which was not certified by peer review) is the author/funder, who has granted bioRxiv a license to display the preprint in perpetuity. It is made available under aCC-BY 4.0 International license.

# Supplementary Figure 5

A

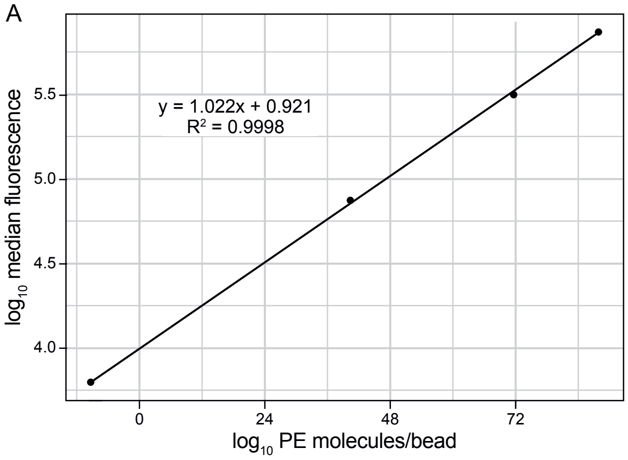

Supplement: Supplement 4 [file NIHPP2026.03.11.711103v1-supplement-4.pdf]
